# The effects of thymic stromal lymphopoietin and IL-3 on human eosinophil–basophil lineage commitment: Relevance to atopic sensitization

**DOI:** 10.1002/iid3.20

**Published:** 2014-05-09

**Authors:** Claudia C K Hui, Sina Rusta-Sallehy, Ilan Asher, Delia Heroux, Judah A Denburg

**Affiliations:** Division of Allergy & Clinical Immunology, Department of Medicine, McMaster UniversityHamilton, Ontario, Canada

**Keywords:** Atopy, eosinophil–basophil progenitors, hemopoiesis, IL-3, TSLP

## Abstract

An important immunopathological hallmark of allergic disease is tissue eosinophilic and basophilic inflammation, a phenomenon which originates from hemopoietic progenitors (HP). The fate of HP is determined by local inflammatory cytokines that permit “in situ hemopoiesis,” which leads to the accumulation of eosinophils and basophils (Eo/B). Given that recent evidence supports a critical immunomodulatory role for thymic stromal lymphopoietin (TSLP) in allergic inflammation, as well as TSLP effects on CD34+ progenitor cytokine and chemokine secretion, we investigated the role of TSLP in mediating eosinophilo- and basophilopoiesis, the mechanisms involved, and the association of these processes with atopic sensitisation. In the studies presented herein, we demonstrate a direct role for TSLP in Eo/B differentiation from human peripheral blood CD34+ cells. In the presence of IL-3, TSLP significantly promoted the formation of Eo/B colony forming units (CFU) (including both eosinophils and basophils) from human HP (HHP), which was dependent on TSLP–TSLPR interactions. IL-3/TSLP-stimulated HHP actively secreted an array of cytokines/chemokines, key among which was TNFα, which, together with IL-3, enhanced surface expression of TSLPR. Moreover, pre-stimulation of HHP with IL-3/TNFα further promoted TSLP-dependent Eo/B CFU formation. HHP isolated from atopic individuals were functionally and phenotypically more responsive to TSLP than those from nonatopic individuals. This is the first study to demonstrate enhanced TSLP-mediated hemopoiesis ex vivo in relation to clinical atopic status. The capacity of HHP to participate in TSLP-driven allergic inflammation points to the potential importance of “in situ hemopoiesis” in allergic inflammation initiated at the epithelial surface.

## Introduction

The airway epithelium is an important initiator of the allergic response; it secretes cytokines/chemokines, which regulate innate immune cells [1]. One such cytokine is thymic stromal lymphopoietin (TSLP), an IL-7-like cytokine [2], which can be elicited from human airway epithelial cells by cytokines and pathogen-associated molecular patterns [3,4]. Many effector cells involved in allergic diseases such as eosinophils [5], basophils [6,7], and mast cells [3,8] have all been shown to respond to TSLP with increased survival, differentiation, and cytokine secretion. TSLP signals through a heterodimeric receptor complex consisting of the IL-7Rα chain and a TSLP binding chain (TSLPR) [9]. TSLP is known to signal through the JAK/STAT and MAPK pathways [10,11], both of which are involved in the differentiation of hemopoietic progenitor cells into eosinophils [12,13]. However, the pathways involved in basophilopoiesis remain unclear [7]. Nonetheless, human eosinophils and basophils are closely linked during development and share a common progenitor [14–16], while in the mouse these differentiative pathways appear distinct [17].

Human eosinophils and basophils differentiate from a common *committed* CD34+ hemopoietic progenitor cell, the eosinophil–basophil (Eo/B) progenitor, found in bone marrow, cord blood, and peripheral blood (PB) [14]. We have previously provided evidence that allergic inflammation is, at least in part, a result of CD34+ progenitors homing to sites of inflammation where they differentiate, under the control of local inflammatory cytokines, into eosinophils and basophils, a process referred to as “in situ hemopoiesis” [18–20]. This overarching concept is supported of findings of many investigators: Siracusa et al. [6], demonstrated that cytokines found at sites of inflammation (IL-3 or TSLP) can differentially impact the differentiation of murine progenitors into effector cells (basophils), resulting in functional and phenotypic heterogeneity; Sergejeva et al. [21], reported that ∼10% of the eosinophilic cells found in murine bronchial alveolar lavage fluid post-allergen exposure was derived from eosinophil-lineage committed precursor cells, or local production of eosinophils within the airway; Robinson et al. [22], Kim et al. [23], and Dorman et al. [24] collectively showed that human CD34+ progenitors are detected in the bronchial and nasal mucosa, and sputum, respectively, of patients with atopic asthma and nasal polyposis, with increased numbers of CD34+/IL-5Rα+ cells found in the airways and sputum of asthmatics following allergen challenge, suggesting that CD34+Eo/B lineage committed cells are found in the tissue [22,24]; furthermore, Allakhverdi et al. [8] demonstrated that human CD34+ progenitors can be induced by TSLP to produce Th2 cytokines, principally IL-5 and IL-13, and that these double-positive CD34+ cells are present in sputum after airway allergen challenge of atopic asthmatics, suggesting that progenitors may act as proinflammatory effector cells and directly contribute to allergic inflammation.

Recent evidence supports a critical immunomodulatory role for TSLP in allergic inflammation, as well as TSLP effects on CD34+ progenitor cytokine and chemokine secretion [8], but the biological effects of TSLP on human PB CD34+ progenitor Eo/B lineage commitment have not been previously described. In this study, we examine the influence of TSLP on IL-3-dependent CD34+ progenitor differentiation via phenotypic and functional human hemopoietic progenitor (HHP)-related Eo/B lineage commitment. Additionally, we elucidate the mechanisms through which TSLP enhances IL-3-mediated eosinophilo- and basophilopoiesis, and the association of these processes with atopic sensitisation.

## Methods

### Subjects

This study was approved by the Hamilton Health Sciences Research Ethics Board (approval number 08-015) and all subjects provided written informed consent. Atopy-unattributable subjects were initially recruited for the study (Figs. 4), following which, subjects with (*n* = 10) or without (*n* = 10) atopy were recruited (Fig. 5). Atopy was defined as a positive skin prick test response (>2-mm wheal) to at least one of 14 common aeroallergens. Further subject characteristics are shown in Table1.

**Figure 1:**
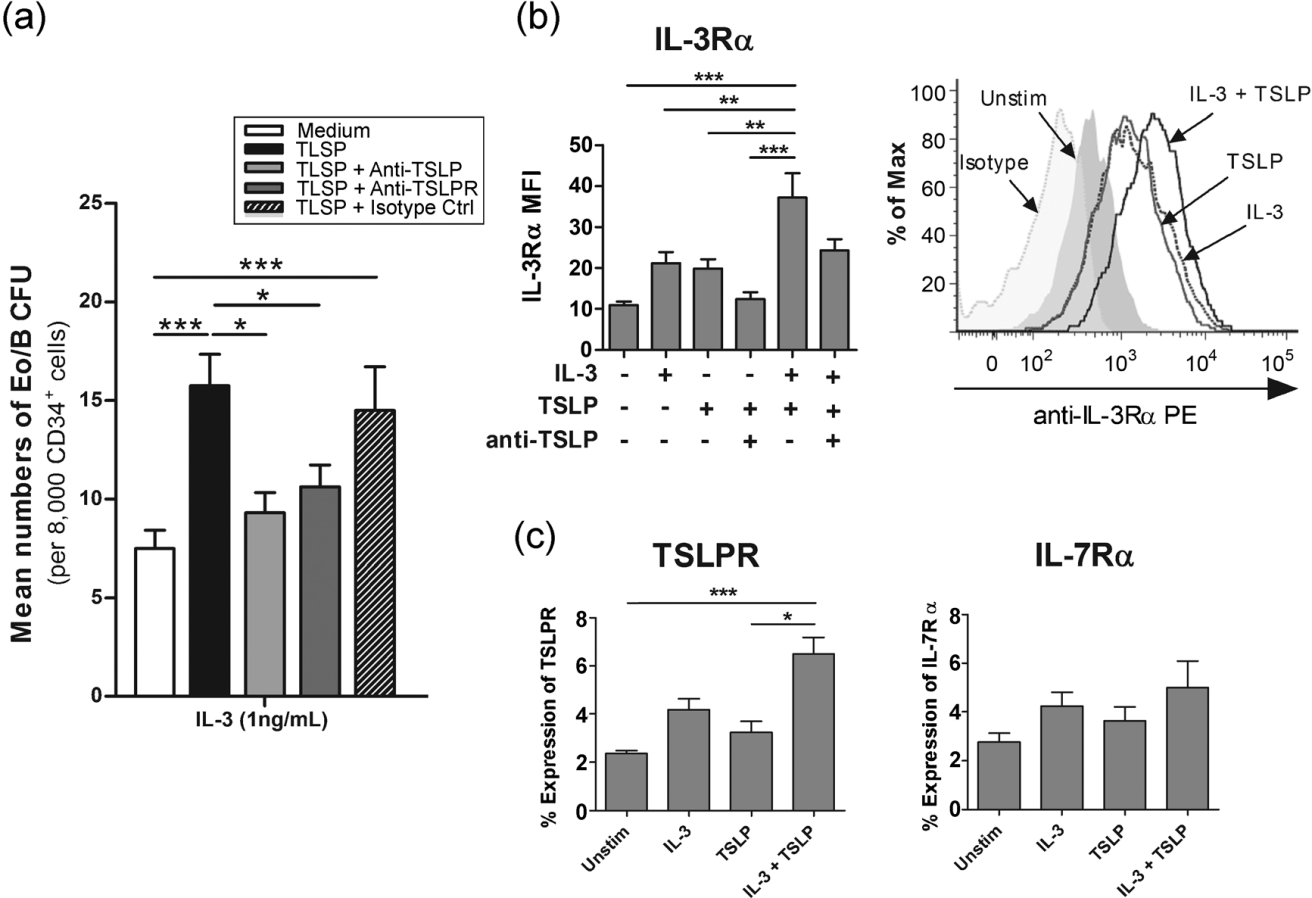
IL-3 and TSLP mediate Eo/B differentiation from PB HHP. PB CD34+ cells were stimulated as indicated and assessed for (a) Eo/B CFU by methylcellulose cultures (*n* = 8 in duplicates) and surface expression of (b) IL-3Rα (*n* = 6), and (c) TSLPR (*n* = 8) and IL-7Rα (*n* = 7) by flow cytometry. (b) A representative overlay histogram for expression of IL-3Rα on unstimulated (shaded gray), IL-3 (dotted line), TSLP (gray line), and IL-3/TSLP (black line) stimulated CD34+ cells. Normal mouse IgG was used as an isotype control (shaded-light gray). Percent expression is the percent of CD34+ cells expressing a given antigen at the 98% confidence limit (i.e., relative to a quadrant marker set to include 2% of cells stained with isotype control antibody). Results shown are mean ± SEM. One independent experiment performed per subject. *P < 0.05; **P < 0.01; ***P < 0.001.

**Figure 2:**
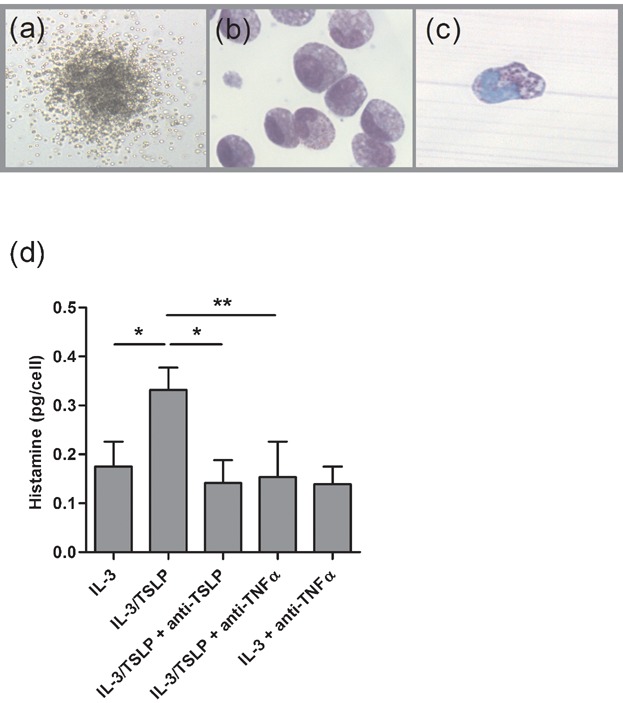
TSLP enhances IL-3-induced basophilopoiesis from PB HHP. (a) Representative pictograph of a colony on day 14 of a methylcellulose colony culture, which was then plucked and cytospin preparations made and stained for (b) eosinophils and (c) basophils with Diff-Quik solution and toluidine blue respectively. (d) Histamine assay was performed on individual colonies on day 14 of methylcellulose colony cultures (*n* = 15). Results shown are mean ± SEM. One independent experiment performed per subject.*P < 0.05; **P < 0.01.

**Figure 3:**
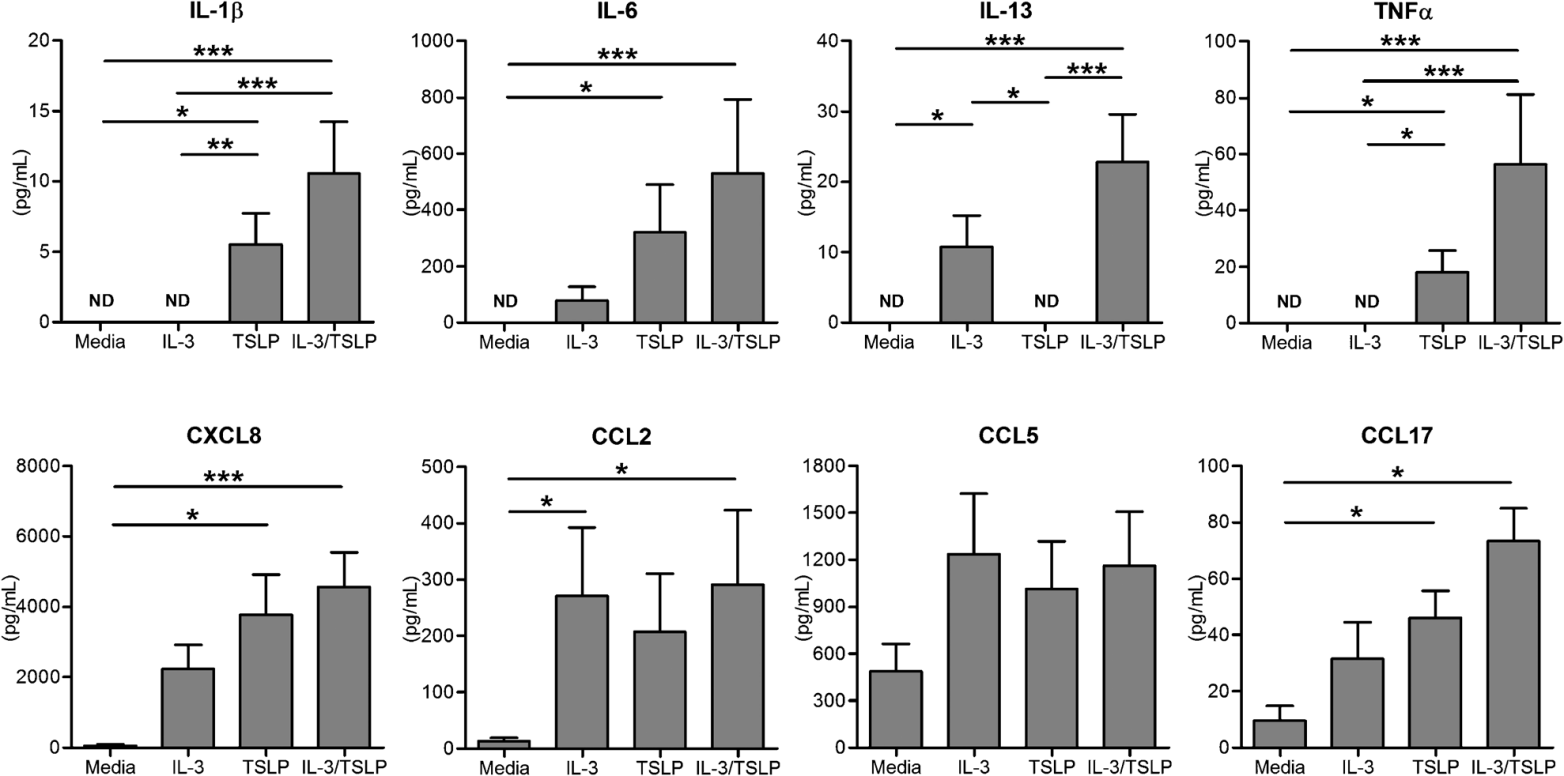
IL-3 and TSLP increase cytokine and chemokine secretion by PB HHP. PB CD34+ cells were stimulated overnight as indicated and cell-free supernatants were collected and measured for cytokine/chemokine secretion (pg/mL) using Luminex. Results shown are mean ± SEM in duplicates (*n* = 8). One independent experiment performed per subject. *P < 0.05; **P < 0.01; ***P < 0.001. ND; not detected.

**Figure 4:**
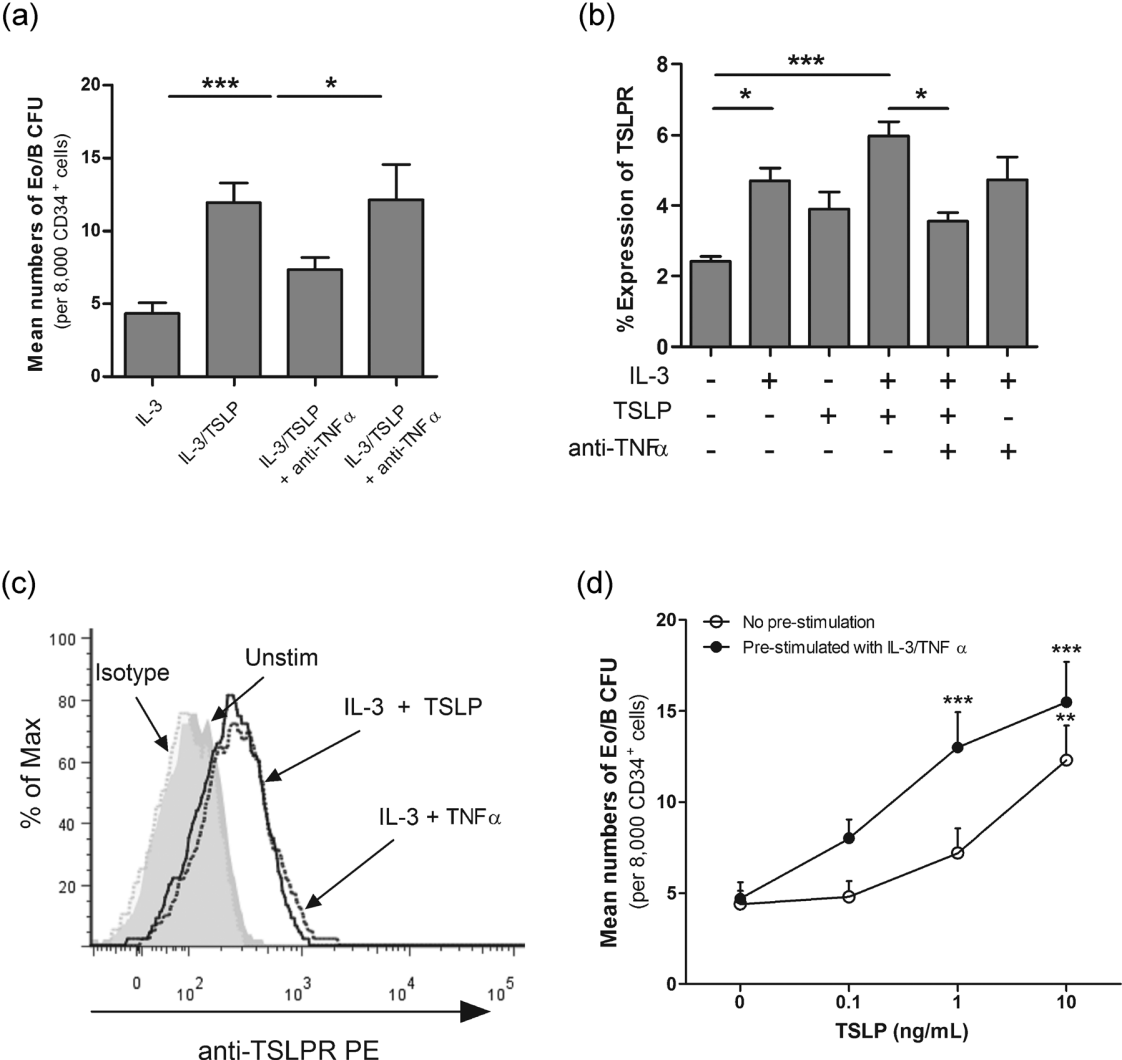
IL-3 and TNFα increase TSLPR expression and sensitivity of PB HHP to TSLP. PB CD34+ cells were treated with IL-3 (1 ng/mL), TSLP (10 ng/mL), and anti-TNFα (10 µg/mL). (a) Eo/B CFU (defined as tight, granular clusters ≥40 cells) were enumerated at the end of 14 days methylcellulose cultures (*n* = 6 in duplicates). (b) surface expression of TSLPR on PB CD34+ cells were analyzed by flow cytometry following 24 h stimulation (*n* = 7). (c) A representative overlay histogram for expression of TSLPR on unstimulated (shaded gray), IL-3 and TNFα (gray line) and IL-3 and TSLP (black line) stimulated CD34+ cells. Normal mouse IgG was used as an isotype control (shaded-light gray). Percent expression is the percent of CD34+ cells expressing a given antigen at the 98% confidence limit (i.e., relative to a quadrant marker set to include 2% of cells stained with isotype control antibody). This experiment was performed four independent times with similar results. (d) PB CD34+ cells were pre-stimulated with IL-3 (1 ng/mL) and TNFα (50 pg/mL) for 24 h and cultured in methylcellulose colony assays in the presence of IL-3 (1 ng/mL) and increasing doses of TSLP (0.1, 1, 10 ng/mL). On day 14, Eo/B CFU were enumerated (*n* = 5 in duplicates, **P < 0.01; ***P < 0.001 compared with TSLP (0 ng/mL) within group). Results shown are mean ± SEM. One independent experiment performed per subject. *P < 0.05; **P < 0.01; ***P < 0.001

**Figure 5:**
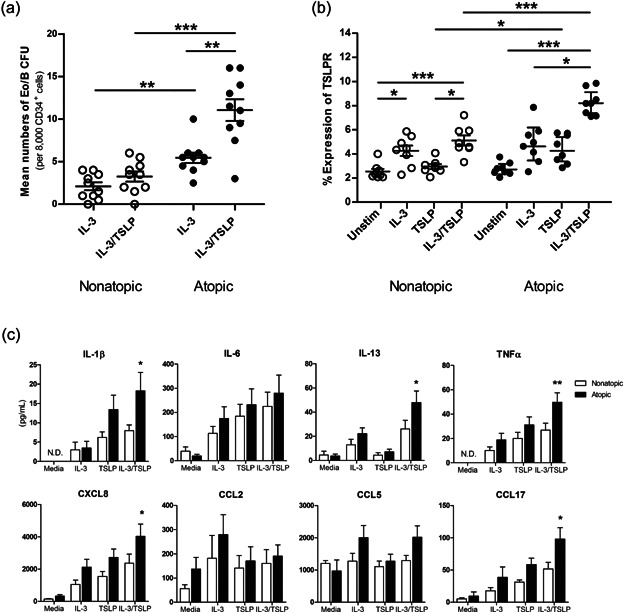
Differential Eo/B CFU, TSLPR expression, and cytokine/chemokine secretion from PB HHP from nonatopic and atopic individuals. PB CD34+ cells from nonatopic (open circles) and atopic (closed circles) subjects were stimulated as indicated and assessed for (a) Eo/B CFU by methylcellulose cultures (*n* = 10 in duplicates), (b) surface expression of TSLPR by flow cytometry (*n* = 8), and (c) cytokine/chemokine secretion by Luminex (*n* = 8 in duplicates). Percent expression is the percent of CD34+ cells expressing a given antigen at the 98% confidence limit (i.e., relative to a quadrant marker set to include 2% of cells stained with isotype control antibody). Results shown are mean ± SEM. One independent experiment performed per subject. *P < 0.05; **P < 0.01; ***P < 0.001. ND; not detected.

**Table:** Subject demographics

Characteristics	Nonatopics (*n* = 10)	Atopics (*n* = 10)
Sex		
Male	5 (50)	7 (70)
Female	5 (50)	3 (30)
Age		
18 to <30	6 (60)	4 (40)
30 to <60	3 (30)	6 (60)
≥60	1 (10)	0
Mean*	29.9 (13.6)	28.5 (7.4)

Values in parentheses indicate percentages.

* Mean (SD).

### Blood collection and processing

One hundred mL of blood were collected through direct venipuncture into heparinized vacutainer tubes (Becton–Dickinson, Franklin Lakes, NJ, USA). Peripheral blood mononuclear cells were isolated by density centrifugation and CD34+ progenitors were enriched using EasySep™ Human Progenitor Cell Enrichment Kit with Platelet Depletion (STEMCELL Technologies, Vancouver, BC, Canada) as per manufacturer's instructions.

### Methylcellulose colony assays

Enriched CD34+ progenitors (8000 cells/well) were cultured in duplicates in 0.9% methylcellulose (Sigma Aldrich, St. Louis, MO, USA) with Iscove's 2+ (modified Dulbecco's medium (Gibco, Burlington, Ontario, Canada) supplemented with FBS, penicillin–streptomycin, and 2-ME) and IL-3 (1 ng/mL), IL-5 (1 ng/mL), or GM-CSF (10 ng/mL; BD Biosciences, Mississauga, ON, Canada) in the presence or absence of TSLP (10 ng/mL; PeproTech, Rocky Hill, NJ, USA) in 12-well plates (Corning Costar, Corning, NY, USA). In some experiments, cells were treated with anti-TSLP (Amgen, Seattle, WA, USA), anti-TSLPR (R&D Systems, Minneapolis, MN, USA), anti-TNFα (R&D), or isotype control (each at 10 µg/mL). Treatment with the indicated stimulatory/inhibitory conditions had no effects on cell viability as determined by trypan blue exclusion. Cultures were incubated for 14 days (37°C, 5% CO_2_). Eo/B CFU were enumerated using inverted light microscopy (colonies were defined as tight, granular clusters ≥40 cells).

### Antibodies

Antibodies used included CD123-PE, TSLPR-PE, IL-7Rα-APC, CD34-PerCP, and CD45-eFluor 450. IL-3R and TSLPR/IL-7Rα and their respective isotype controls were purchased from BD Biosciences, Mississauga, ON, Canada and eBioscience, San Diego, CA, USA, respectively. CD34 and CD45 antibodies were purchased from BioLegend and eBioscience, San Diego, CA, USA, respectively.

### Cell staining

Isolated CD34+ progenitors (10^6^ cells/mL) were stimulated overnight as indicated. Following which, CD34+ progenitors were stained as previously described with modification [18]. Briefly, cells were washed with fluorescence-activated cell sorting buffer (PBS containing 0.1% sodium azide) and resuspended in murine block (1 × 10^5^ cells/tube) and incubated in the dark (15 min, 4°C). Next, optimal amounts of isotype controls or test antibodies were added and incubated in the dark (30 min, 4°C). Cells were then washed, fixed in cytofix (BD), and stored in the dark at 4°C until ready for acquisition.

### Acquisition and analysis

Stained cells were acquired with a LSR II flow cytometer (BD Biosciences) using the FACSDiva software (BD Biosciences). Offline analysis was performed using FlowJo software (Tree Star, Ashland, OR, USA). CD34+ cells were enumerated using a previously established multi-parameter sequential gating strategy [18]. CD34+ progenitor cells were identified as having high CD34 expression, low-intermediate CD45 expression, and low forward and side scatter (Supplementary Fig. S1). Receptor expression data were collected as the percentage of positive cells at the 98% confidence limit (i.e., relative to a quadrant marker set to include 2% of cells stained with isotype control antibody). Median fluorescence intensity (MFI) is defined as the MFI of the receptor of interest divided by the MFI of the isotype control. Due to the use of an enrichment protocol, absolute numbers of CD34+ cells were not determined.

### Cytokine and chemokine secretion

CD34+ cells (10^6^ cells/mL) were stimulated overnight as indicated and cell-free supernatant was harvested and IL-1β, IL-4, IL-6, IL-9, IL-10, IL-13, GM-CSF, IFNγ, TNFα, CXCL8, eotaxin, CCL2 (MCP-1), CCL5 (RANTES), and CCL17 (TARC) were assessed using Bio-Plex assays (Bio-Rad, Hercules, CA, USA) according to manufacturer's recommendation. The detection limits for these cytokines were 3.2 (IL-1β), 2.2 (IL-4), 2.3 (IL-6), 2.1 (IL-9), 2.2 (IL-10), 3.7 (IL-13), 2.2 (GM-CSF), 92.6 (IFNγ), 5.8 (TNFα), 1.0 (CXCL8), 40.9 (eotaxin), 2.1 (CCL2), 2.2 (CCL5), and 1.7 pg/mL (CCL17).

### Histochemical stains

Individual Eo/B CFU cells were picked from methylcellulose and placed into PBS. Cytospin preparations were made on glass slides using Shandon Cytocentrifuge 3 (Shandon Southern Instruments, Cambridge, UK). Eosinophils were identified using Diff-Quik (Siemens, Erlangen, Germany) and basophils identified using toluidine blue stain (Sigma).

### Histamine assay

The total number of cells in each individual colony was enumerated using inverted light microscopy before being picked from methylcellulose and placed into PBS, boiled (99°C, 5 min), centrifuged, and cell-free supernatant harvested and measured for histamine content using Histamine Enzyme Immunoassay Kit (Bertin Pharma, Montigny-le-Bretonneux, France) according to manufacturer's recommendation. The detection limit of this assay is 55 pg/mL.

### Statistical analysis

All data are expressed as the mean ± SEM. Significance was assumed at *P* < 0.05. All analyses were performed with Prism version 5 (GraphPad Software, La Jolla, CA, USA) using non-parametric tests. Differences within groups were assessed by Friedman test with Dunnett post hoc test. Between-group comparisons (nonatopic vs. atopic) were made using the Mann–Whitney *U*-test.

## Results

### TSLP preferentially enhances IL-3-dependent Eo/B differentiation from PB HHP

The addition of TSLP significantly increased the formation of IL-3-responsive Eo/B CFU from 7.56 ± 0.9 to 15.8 ± 1.6 (*P* < 0.001; Fig. 1a), which was inhibited by the addition of neutralizing anti-TSLP (9.3 ± 1.0 per 8000 CD34+ cells; *P* < 0.05) and anti-TSLPR (10.6 ± 1.1 per 8000 CD34+ cells; *P* < 0.05). TSLP did not have any effects on either IL-5- or GM-CSF-responsive Eo/B CFU (Supplementary Fig. S2). Moreover, effects of TSLP on HHP were not enhanced by IL-33 (data not shown).

Overnight stimulation of HHP with IL-3/TSLP together enhanced the MFI of IL-3Rα compared to unstimulated (3.4-fold; *P* < 0.001) and TSLP-stimulated (1.9-fold; *P* < 0.01; Fig. 1b). Overnight stimulation with IL-3/TSLP significantly increased percent expression of TSLPR compared to unstimulated (*P* < 0.001) and TSLP-stimulated HHP (*P* < 0.05). A similar trend was seen for IL-7Rα expression, although not significant (Fig. 1c). No significant difference in TSLPR expression was observed following IL-5/GM-CSF and/or TSLP stimulation (Supplementary Fig. S3).

### TSLP enhances IL-3-induced basophilopoiesis from PB HHP

Histochemical staining of individual colonies with DiffQuik and toluidine blue revealed the presence of cells with either eosinophilic granular cytoplasm or metachromatic granules, morphologically consistent with eosinophils and basophils, respectively (Fig. 2b,c). To further confirm the presence of basophils, colony histamine assay was performed. Taking colony size (cell number) into account, the mean (±SEM) calculated amount of histamine (pg/cell) was significantly higher in IL-3/TSLP-induced Eo/B CFU compared to IL-3-induced Eo/B CFU (48%; *P* < 0.05). In the presence of neutralizing anti-TSLP or neutralizing anti-TNFα, the histamine content was significantly lower compared to IL-3/TSLP stimulated colonies (57%; *P* < 0.05 and 54%; *P* < 0.01, respectively; Fig. 2d).

### IL-3 and TSLP increase cytokine and chemokine secretion by PB HHP

TSLP alone induced significant levels of IL-1β, IL-6, TNFα, CXCL8, and CCL17 from PB HHP, compared to unstimulated controls (*P* < 0.05; Fig. 3). IL-3/TSLP-stimulated HHP released significant levels of IL-1β, IL-6, IL-13, TNFα, CXCL8, CCL2, and CCL17 compared to unstimulated controls (*P* < 0.001). PB HHP failed to secrete detectable levels of IL-4, IL-9, IL-10, GM-CSF, IFNγ, and eotaxin; while CCL5 was highly secreted by PB HHP cells under all conditions.

### TNFα plays a key role in IL-3/TSLP-mediated Eo/B CFU formation and TSLPR expression

Next, we investigated whether TSLP-induced TNFα could support Eo/B CFU formation given that IL-3 and TNFα have previously been reported to upregulate TSLPR on human eosinophils [5]. The addition of neutralizing anti-TNFα significantly reduced TSLP-induced IL-3-responsive Eo/B CFU (38%; *P* < 0.05; Fig. 4a) and surface expression of TSLPR on HHP (40.1%; *P* < 0.05; Fig. 4b) compared to IL-3/TSLP-stimulated HHP. Overnight stimulation of PB HHP with IL-3 and TNFα (50 pg/mL) increased TSLPR expression to comparable levels post TSLP/IL-3-stimulation (Fig. 4c) and promoted Eo/B CFU formation at lower concentrations of TSLP, which was statistically significant compared to IL-3-stimulated HHP (*P* < 0.001; Fig. 4d).

### PB HHP from atopic individuals exhibit enhanced Eo/B differentiation, TSLPR expression, and cytokine/chemokine secretion

We next examined responses of HHP in relation to atopic sensitization. PB HHP derived from atopic individuals produced significantly higher numbers of Eo/B CFU compared to those from nonatopic individuals following stimulation with IL-3 (5.4 ± 0.6 vs. 2.1 ± 0.5 per 8000 CD34+ cells; *P* < 0.01) and IL-3/TSLP (11.1 ± 1.3 vs. 3.3 ± 0.6 per 8000 CD34+ cells; *P* < 0.001; Fig. 5a). Within the atopic group, TSLP significantly increased the formation of IL-3-responsive Eo/B CFU (twofold; *P* < 0.01; Fig. 5a). No differences in TSLPR expression were observed between nonatopic and atopic individuals at baseline or after IL-3-stimulation (Fig. 5b). However, TSLPR expression was significantly higher in atopic subjects compared to nonatopic subjects following stimulation with TSLP (4.40 ± 0.42% expression vs. 2.95 ± 0.20% expression; *P* < 0.05) and IL-3/TSLP (8.26 ± 0.38% expression vs. 5.10 ± 0.42% expression; *P* < 0.001; Fig. 5b). Furthermore, PB HHP derived from atopic individuals produced significantly higher levels of IL-1β (*P* < 0.05), IL-13 (*P* < 0.05), TNFα (*P* < 0.01), CXCL8 (*P* < 0.05), and CCl17 (*P* < 0.05), compared to PB HHP from nonatopic individuals, post IL-3/TSLP-stimulation (Fig. 5c).

## DISCUSSION

We demonstrate for the first time that PB HHP respond directly to TSLP in vitro with enhanced Eo/B colony formation and TSLPR/IL-7Rα expression, specifically upon co-stimulation with IL-3, and not IL-5 or GM-CSF. This differentiation process appears dependent on autocrine and/or paracrine signaling by TNFα-producing progenitors. IL-3 and TNFα, cytokines which are found at sites of allergic inflammation, may help explain the increase the sensitivity of HHP to TSLP-mediated Eo/B differentiation. Finally, we demonstrate enhanced stimulatory effects of IL-3 and TSLP on PB CD34+ progenitors derived from atopic individuals. To the best of our knowledge, this is the first study to demonstrate such findings in humans ex vivo. These findings provide a novel mechanism underlying eosinophil and basophil accumulation in tissues during allergic inflammation, linked as it is to TSLP and its known amplification of Th2 immune responses in atopic individuals.

TSLP–TSLPR interactions are crucial to the development of eosinophilia [25] and basophilia [6] in mice; however, its role in humans is unclear. We show for the first time the importance of TSLP–TSLPR in Eo/B lineage commitment of IL-3-responsive HHP. Our group and others have previously reported the presence of both eosinophils and basophils in IL-3-stimulated hemopoietic progenitor cultures [14–16]; our current findings therefore demonstrate that TSLP can serve the role of a key epithelial-derived factor in this process of human Eo/B differentiation. Differential counts of colony cells were not formally performed; therefore, relative proportions of eosinophils versus basophils in these colonies are unclear. However, methylcellulose colony assays have been used for many years by us and others, to both enumerate and assess progenitors and their progeny in response to hemopoietic cytokines (and other stimuli), as quantitated by Eo/B CFU [14,19,26]. We have elected to use the CFU assay, and add histamine assays, as previously extensively documented by us and others, to represent a specific, surrogate biomarker of basophil “content” within these Eo/B colonies, which are each derived from a single progenitor, given that histidine decarboxylase is only present within the basophil, not the eosinophil in these mixed basophil–eosinophil colonies [14,20,27–29]. Since cells within these Eo/B colonies are rather immature and often possess dual phenotypes – including cells with “hybrid” eosinophilic-basophilic granulation by standard morphological–histochemical assessments – differentiating the cells using histochemical stains is not reliable. When the latter are routinely performed, both toluidine blue-positive granules as well as eosinophilic staining granules are found in many cells simultaneously [14–16]. As such, our key (and to our judgment, more robust) arbiter of basophil content remains the colony histamine content. Indeed, our group has previously reported on the close correlation between the basophil numbers and the histamine content in Eo/B CFU [14,20,30]. Additionally, in the current study, the histamine assay allowed for comparative analyses, demonstrating marked differences in histamine content between colonies grown in the presence or absence of TSLP and/or neutralizing anti-TSLP or anti-TNFα. While further analyses are required to examine precisely how TSLP may alter colony histamine content, it appears unique among the epithelial-derived cytokines in its ability to promote basophilia in peripheral tissues [6]. Of note, Siracusa et al. [6] recently reported the ability of TSLP to induce IL-3-*independent* basophils from bone marrow-resident precursors in mice. However, we were unable to observe TSLP alone-mediated Eo/B CFU, suggesting that TSLP must work in concert with IL-3 to induce Eo/B differentiation. We speculate that this discrepancy may reflect inter-species differences in hemopoiesis due to the distinct surface phenotype of progenitors in mice and humans [31], or to the distinct differentiative pathways of eosinophil and basophil development in humans and mice [14,17], or to a combination of these factors. As such, rather than examining the relative proportions of eosinophils versus basophils in these colonies of nascent eosinophils and basophils of mixed granulation (which one also sees in liquid cultures), we have focused on PB Eo/B CFU (and thus, HHP) production after TSLP stimulation, providing novel evidence that TSLPR engagement on PB CD34+ cells has the capacity to enhance Eo/B lineage priming of myeloid progenitors, increasing the likelihood of development of allergic eosinophilic/basophilic inflammation, in addition to disease maintenance or progression.

Allakhverdi et al. recently demonstrated the ability of TSLP, together with IL-33, to promote “Th2-like” properties in human CD34+ progenitors, based on induction of Th2 cytokines (IL-6, IL-13, GM-CSF) and chemokines (CXCL8, CCL1, CCL17, CCL12) [8]. Likewise, we detected levels of IL-1β, IL-6, IL-13, TNFα, CXCL8, CCL2, CCL5, and CCL17 following overnight stimulation of PB HHP with TSLP; IL-4, IL-9, IL-10, GM-CSF, IFNγ, and eotaxin were undetected under all conditions. However, contrary to findings by Allakhverdi et al. [8], we were unable to detect levels of GM-CSF in the supernatant and of the cytokines detected, the concentrations were comparatively low. The higher levels of cytokines reported by Allakhverdi et al. may be due to the use of stem cell factor (100 ng/mL) in their culture medium [32]. Of the Th2 cytokines and chemokines detected, of importance is the enhanced secretion of TNF-α, which, in conjunction with TSLP/IL-33, has been previously shown to enhance cytokine secretion by human cord blood and PB-derived CD34+ progenitors [8]. Furthermore, Caux et al. [33] showed enhancing effects of TNFα on IL-3- and GM-CSF-dependent proliferation of CD34+ HHP. It is plausible that IL-3/TSLP-induced TNFα is a key event in HHP autocrine secretion of Th2-like cytokines/chemokines and in Eo/B differentiation. Our observation that inhibiting TNFα reduces TSLPR expression on HHP, which is in agreement with a study that showed enhanced TSLPR expression on mature eosinophils post IL-3/TNFα-stimulation [5], may explain the reduction in Eo/B CFU formation and colony histamine levels in our cultures. The relevance of TNFα antagonists in decreasing eosinophil and/or basophil counts in vivo is unclear; however, anti-TNFα agents have been reported to decrease sputum histamine levels and improve asthma outcomes, airway hyper-responsiveness, and exacerbation rates [34,35]. Furthermore, in OVA-sensitized allergic rhinitis and bleornycin-induced pulmonary fibrosis murine models, TNFα antagonists have been shown to inhibit eosinophilia in the nasal mucosa and lung, respectively [36,37].

Although we have not herein reported on which signaling pathways are involved in TSLP-induced Eo/B differentiation, we do have findings implicating the preferential dependence of p38MAPK signaling pathways in IL-3/TSLP-mediated Eo/B differentiation (Supplementary Fig. S4). Indeed, Fanat et al. [38] showed that supernatants from TNFα-, IL-1β-, and IFNγ–stimulated human airway smooth muscle (HASM) cells drive eosinophilic differentiation from HHP in vitro, in a p38MAPK-dependent way. TNFα/IL-1β have been shown to induce TSLP production from HASM cells [39]; we therefore speculate the preferential dependence of TSLPR/p38MAPK signal transduction in TSLP-mediated Eo/B differentiation [38].

**Figure 6:**
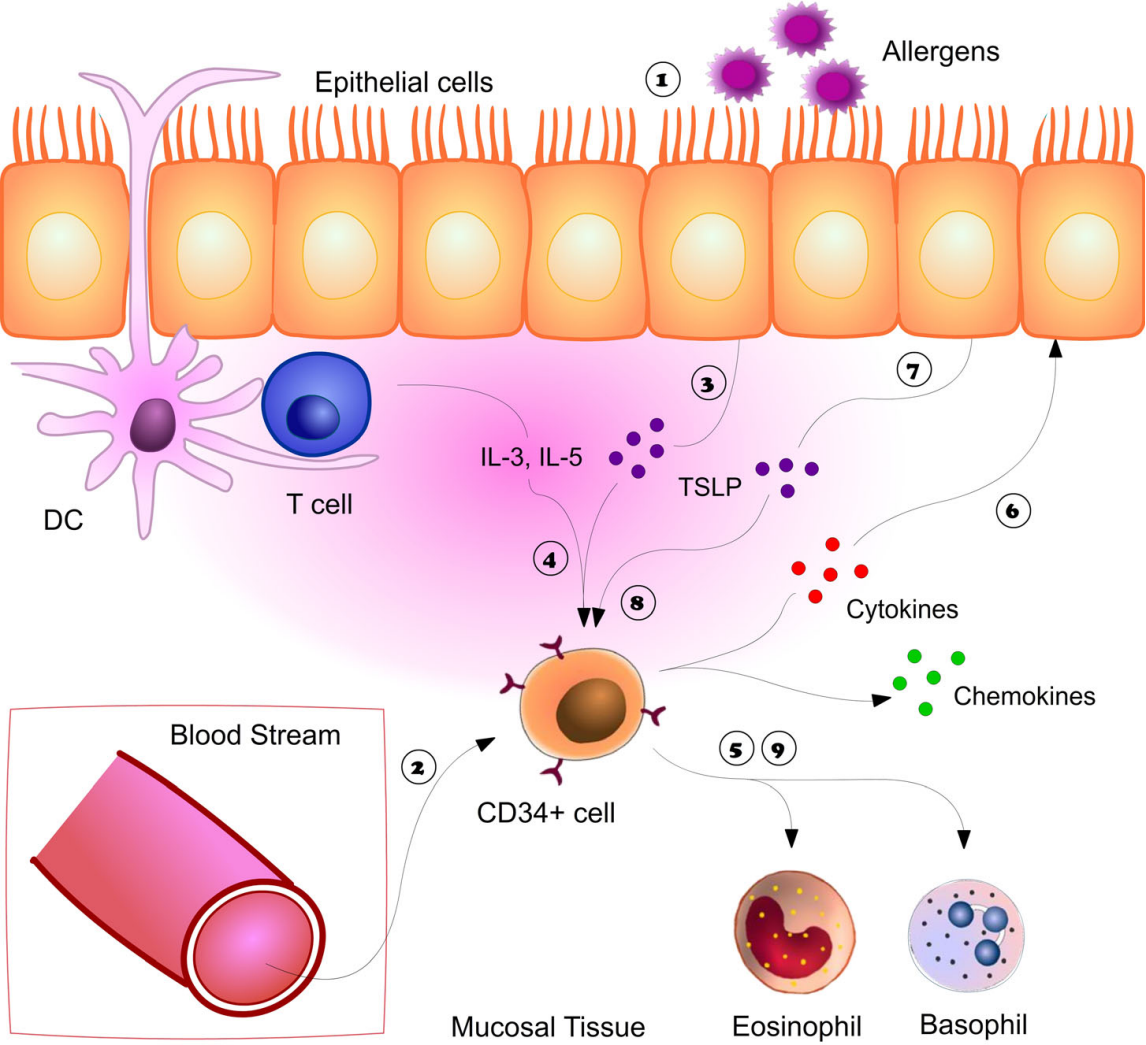
Autocrine/paracrine regulation of TSLP-mediated Eo/B differentiation. Following exposure to allergens 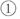, increase CD34+ progenitors are recruited and detected at sites of inflammation 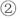. Allergen induce epithelial-derived TSLP 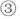 along with local cytokines (IL-3) activate CD34+ cells with release of cytokines and chemokines 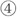 as well as induce in situ hemopoiesis – the differentiation of CD34+ progenitor cells into eosinophils and basophils 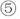. The cytokines secreted by CD34+ cells can directly activate the epithelium 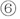 resulting in the release of more TSLP 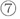, which can act cooperatively with local cytokines and/or CD34+ released cytokines to further enhance eosinophilo/basophilopoiesis in situ 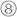, 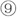.

Atopic sensitization is a widely recognized risk factor for allergic diseases [40]. In the current study, we show that PB HHP from atopic individuals are more responsive to IL-3 and even more so to TSLP, resulting in elevated numbers of Eo/B CFU post IL-3/TSLP-stimulation, compared to HHP from nonatopic individuals. This novel finding related to TSLP effects is in keeping with previous observations that atopic sensitization and disease are associated with enhanced PB (and bone marrow) HHP Eo/B lineage commitment and tissue allergic inflammation related to more “classic” eosinophilopoietic cytokines such as IL-5 [18,22,23,26]. Furthermore, our group has demonstrated increased levels of IL-5-responsive progenitors in atopic subjects, compared with nonatopic subjects [18]. Likewise, the observation that HHP from atopic individuals respond more robustly to TSLP/IL-3 may be related to increased levels of IL-3-responsive progenitors. Alternatively, PB HHP from atopic subjects may have increased endogenous expression of IL-3, with consequent autocrine effects on Eo/B differentiation, as Kuo et al. [41] have shown with IL-5. The increased TSLP responsiveness of HHP from atopic individuals may be a reflection of increased production of TNFα compared to HHP from nonatopic individuals (Fig.6 c). We show in the current study that TNFα, together with IL-3, enhance TSLPR expression and HHP sensitivity to TSLP-mediated Eo/B differentiation. These findings are consistent with an atopic ‘priming’ effect on HHP, such that they differentiate into Eo/B CFU more readily to IL-3/TSLP than HHP from nonatopic individuals, contributing to the development of tissue eosinophilia/basophilia. Of note, given the lack of any significant effects of TSLP on IL-5- and GM-CSF-Eo/B differentiation in non-attributable PB HHP (Fig. S2), we did not examine the combination of IL-5/TSLP nor GM-CSF/TSLP in the HHP of known atopic individuals, but rather concentrated on the TSLP/IL-3-mediated pathway.

There is also documented increased secretion of TSLP from bronchial epithelial cells of atopic, versus nonatopic individuals [4], suggesting that TSLP may play an important tissue role, such as enhancement of “in situ hemopoiesis” in atopy. Although the precise mechanisms are unclear, we postulate paracrine effects of epithelial cells on tissue-resident HHP, which have been detected at mucosal sites of allergic inflammation [22,23]. We demonstrate in the current study that IL-3/TSLP induces TNFα and IL-1β secretion, previously shown to mediate TSLP secretion from airway epithelial cells [3]. Moreover, our data illustrate that pre-stimulation of PB CD34+ cells with IL-3 and TNFα upregulates TSLPR expression, which significantly increases the sensitivity of CD34+ cells to TSLP-mediated Eo/B differentiation. It is possible that epithelial-derived TSLP, along with local hemopoietic factors (i.e., IL-3), both of which are elevated in atopic individuals [4,42], may target HHP resident at mucosal sites, triggering the paracrine and/or autocrine differentiation of these recruited progenitor cells (Fig. 6). TNFα can be produced by various cell types other than CD34+ progenitors; however, in the current study, CD34+ progenitors were enriched using a protocol which virtually eliminated all T- and B-cells.

Although flow cytometry was not performed to check for residual T cells following separation, the ability of CD34+ cells themselves to produce Th2-like cytokines and TNFα following TSLP stimulation and toll-like receptor-ligation has previously been reported [3,43].

Finally, the preferential synergy of TSLP with IL-3, and not IL-5 or GM-CSF, in driving Eo/B differentiation is consistent with previous work which demonstrates that the responsiveness of progenitors to these cytokines is highly dependent on the stage of differentiation [44,45]. PB CD34+ cells consist of mainly immature progenitors, and therefore mostly respond to IL-3 and GM-CSF, early-acting cytokines [44,46], which is consistent with our findings (Fig. 1a and Supplementary Fig. S2). Of note, IL-3 tends to drive CD34+ progenitor differentiation primarily along the basophil-lineage [47], while GM-CSF promotes the differentiation of a mixed population (Eo/B cells, macrophages, etc.) [46]. Therefore, the preferential differentiation of PB CD34+ progenitors to IL-3-responsive lineage suggests that the response of PB HHP to TSLP involves basophil-mediated inflammation [6].

In summary, our study uncovers a previously unrecognized role for TSLP in allergic inflammation – as a regulator of in situ hemopoiesis, by enhancing the process of HHP (CD34+ cell) differentiation within tissues. The enhanced TSLP-mediated hemopoiesis ex vivo in relation to clinical atopic status may help explain increased eosinophils and basophils at sites of inflammation in atopic individuals.

## SUPPORTING INFORMATION

**Figure S1.** Flow cytometric multi-gating strategy for receptor expression on PB CD34+ cells. **Figure S2.** TSLP has no effect on IL-5- and GM-CSF-responsive Eo/B CFU. **Figure S3.** IL-5 and GM-CSF has no effect on TSLPR expression. **Figure S4.** p38MAPK signal transduction is preferentially involved in TSLP-mediated Eo/B differentiation.
