# Supplementary material for: The effects of thymic stromal lymphopoietin and IL-3 on human eosinophil–basophil lineage commitment: Relevance to atopic sensitization
Source: Immun Inflamm Dis. 2014 May 9;2(1):44–55. doi: 10.1002/iid3.20 (PMC4220668; doi:10.1002/iid3.20)
Supplement: Supplementary file 1 [file iid30002-0044-SD1.docx]

**Supplementary Figure Legends**

**Fig. S1. Flow cytometric multi-gating strategy for receptor expression on PB CD34+ cells.** Gating was based on high CD34 and low-intermediate CD45 positivity combined with low forward and side scatter, characteristics of CD34+ progenitor cells. (a) An initial gate (R1) was set to capture CD45+ cells (leukocytes). (b) A subsequent gate was made to capture CD34-bright cells (R2). (c) R3 was then gated to capture CD45+ cells with mononuclear morphology. (d) Cells with low forward and side scatter, consistent with mononuclear cell morphology, was gated (R4). Finally, cells in R4 (true CD34+ population) were further analyzed for staining with PE-linked TSLPR (f) or isotype control (e). Receptor expression data were collected as the percentage of positive cells at the 98% confidence limit (ie. relative to a quadrant marker set to include 2% of cells stained with isotype control antibody).

**Fig. S2. TSLP has no effect on IL-5- and GM-CSF-**responsive **Eo/B CFU.** PB CD34+ cells were stimulated with (a) IL-5 and (b) GM-CSF and assessed for Eo/B CFU by methylcellulose cultures. Results shown are mean ± SEM in duplicates (n=5). One independent experiment performed per subject.

**Fig. S3. IL-5 and GM-CSF has no effect on TSLPR Expression.** PB CD34+ cells were stimulated as indicated to examine the effects of (a) IL-5 (n=5) and (b) GM-CSF (n=4) on TSLPR expression. Results shown are mean ± SEM. One independent experiment performed per subject.

**Fig. S4. p38MAPK signal transduction is preferentially involved in TSLP-mediated Eo/B differentiation**. PB CD34+ cells were incubated with STAT5 inhibitor (50µM), U0126 (ERK1/2 inhibitor; 10µM), SB203580 (p38MAPK inhibitor; 10µM), or SP600215 (JNK inhibitor; 50nM) for 1 h before stimulating with IL-3 (1ng/mL) and/or TSLP (10ng/mL). Eo/B CFU (defined as tight, granular clusters ≥40 cells) were enumerated at the end of 14 d methylcellulose cultures. Results shown are mean ± SEM in duplicates (n=6). One independent experiment performed per subject. * P<0.05; ** P<0.01; *** P<0.001.

**Supplementary Figures**


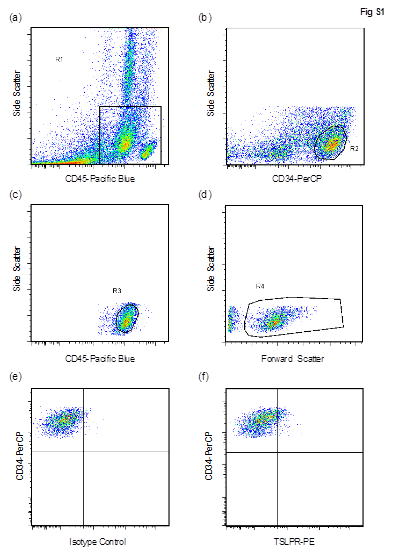


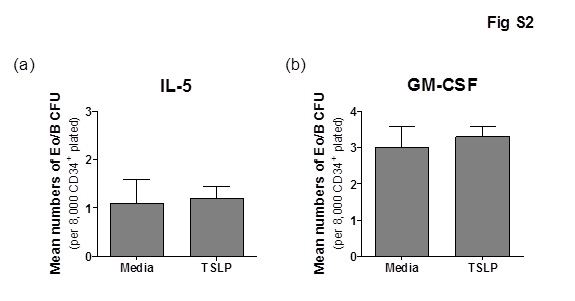


**
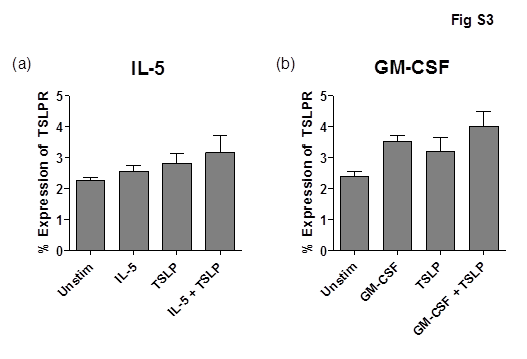
**

**
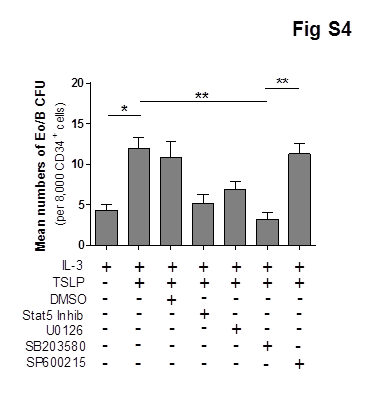
**
